# Diagnostic Accuracy of a Portable Electromyography and Electrocardiography Device to Measure Sleep Bruxism in a Sleep Apnea Population: A Comparative Study

**DOI:** 10.3390/clockssleep5040047

**Published:** 2023-11-20

**Authors:** Rosana Cid-Verdejo, Adelaida A. Domínguez Gordillo, Eleuterio A. Sánchez-Romero, Ignacio Ardizone García, Francisco J. Martínez Orozco

**Affiliations:** 1 Faculty of Dentistry, Universidad Complutense de Madrid, 28040 Madrid, Spain; adelaida@odon.ucm.es (A.A.D.G.); ignacioa@ucm.es (I.A.G.); 2 Department of Clinical Dentistry, Faculty of Biomedical Sciences, Universidad Europea de Madrid, 28670 Madrid, Spain; 3 Interdisciplinary Group on Musculoskeletal Disorders, Faculty of Sport Sciences, Universidad Europea de Madrid, 28670 Villaviciosa de Odón, Spain; 4 Department of Physiotherapy, Faculty of Sport Sciences, Universidad Europea de Madrid, 28670 Villaviciosa de Odón, Spain; 5 Physiotherapy and Orofacial Pain Working Group, Sociedad Española de Disfunción Craneomandibular y Dolor Orofacial (SEDCYDO), 28009 Madrid, Spain; 6 Clinical Neurophysiology Department, Sleep Unit, San Carlos University Hospital, 28040 Madrid, Spain; fjmo2002@yahoo.es

**Keywords:** bruxism, electromyography, sleep apnea, polysomnography, sleep bruxism, sleep–wake disorders, electrocardiography, portable device

## Abstract

Background: The gold standard for diagnosing sleep bruxism (SB) and obstructive sleep apnea (OSA) is polysomnography (PSG). However, a final hypermotor muscle activity often occurs after apnea episodes, which can confuse the diagnosis of SB when using portable electromyography (EMG) devices. This study aimed to compare the number of SB episodes obtained from PSG with manual analysis by a sleep expert, and from a manual and automatic analysis of an EMG and electrocardiography (EKG) device, in a population with suspected OSA. Methods: Twenty-two subjects underwent a polysomnographic study with simultaneous recording with the EMG-EKG device. SB episodes and SB index measured with both tools and analyzed manually and automatically were compared. Masticatory muscle activity was scored according to published criteria. Patients were segmented by severity of OSA (mild, moderate, severe) following the American Academy of Sleep Medicine (AASM) criteria. ANOVA and the Bland–Altman plot were used to quantify the agreement between both methods. The concordance was calculated through the intraclass correlation coefficient (ICC). Results: On average, the total events of SB per night in the PSG study were (8.41 ± 0.85), lower than the one obtained with EMG-EKG manual (14.64 ± 0.76) and automatic (22.68 ± 16.02) analysis. The mean number of SB episodes decreases from the non-OSA group to the OSA group with both PSG (5.93 ± 8.64) and EMG-EKG analyses (automatic = 22.47 ± 18.07, manual = 13.93 ± 11.08). However, this decrease was minor in proportion compared to the automatic EMG-EKG analysis mode (from 23.14 to 22.47). The ICC based on the number of SB episodes in the segmented sample by severity degree of OSA along the three tools shows a moderate correlation in the non-OSA (0.61) and mild OSA (0.53) groups. However, it is poorly correlated in the moderate (0.24) and severe (0.23) OSA groups: the EMG-EKG automatic analysis measures 14.27 units more than PSG. The results of the manual EMG-EKG analysis improved this correlation but are not good enough. Conclusions: The results obtained in the PSG manual analysis and those obtained by the EMG-EKG device with automatic and manual analysis for the diagnosis of SB are acceptable but only in patients without OSA or with mild OSA. In patients with moderate or severe OSA, SB diagnosis with portable electromyography devices can be confused due to apneas, and further study is needed to investigate this.

## 1. Introduction

Sleep bruxism is a masticatory muscle activity during sleep that is characterized as rhythmic (phasic) or non-rhythmic (tonic) and is not a movement disorder or a sleep disorder in otherwise healthy individuals [1]. The basic pattern of sleep bruxism (SB) consists of rhythmic activity of masticatory muscles (RMMA). It is a consequence of several changes due to the activation of the autonomic nervous system, such as the increase in heart rate (HR) [2,3]. Nonetheless, there are other motor events associated with SB, for instance, masticatory muscular activity (MMA), that are not fully explained by these mechanisms. It is suggested that SB is mainly the result of pathophysiological and psychological processes [4,5,6,7] and that it has a multifactorial origin [8,9,10]. SB also occurs concomitantly or secondarily to other sleep disorders, such as obstructive sleep apnea (OSA) [11,12,13,14,15,16,17].

OSA consists of recurrent episodes of partial or total upper airway obstruction (apnea–hypopnea events), accompanied by sleep fragmentation caused by arousals and commonly accompanied by snoring [18,19], in addition to other complications (hypertension, arrhythmias, cardiovascular disease, etc.) [20,21]. The gold standard for diagnosing OSA and SB is PSG. However, using portable EMG devices to diagnose SB can be challenging since different studies use varying criteria for neurophysiological analysis [22,23,24,25,26,27,28]. Different authors suggest that SB studied with PSG in patients with OSA usually occurs close to apnea–hypopnea (AH) events [22,29,30,31]. On the other hand, the causal relationship between SB and OSA is still unclear, with different possible cause–effect theories [27]. Additionally, sleep-related oromotor activity (OMA), such as snoring, lip sucking, and swallowing, can also affect EMG results and add to the complexity of diagnosis.

During the analysis of polysomnography (PSG) recordings, differentiating between RMMA, MMA, OMA, and recognized bruxing activity can be challenging. The automatic analysis mode of EMG devices may not always accurately recognize these types of activities, and not all EMG devices offer a manual analysis mode. Moreover, the criteria used for the manual mode of these recordings are not uniform [3,10,22,30,31,32,33,34,35,36,37]. PSG studies in sleep laboratories include electroencephalography (EEG), electrooculogram (EOG), electrocardiogram (EKG), EMG recordings (of the masticatory muscles and tibial muscles), and thoracoabdominal movement recordings. It also includes oronasal flow and oxygen saturation, allowing a definitive evaluation of SB and the detection of other disorders such as OSA. The apnea–hypopnea index (AHI) can be calculated based on these recordings, which can help categorize the severity of OSA. PSG studies can also aid in the detection of other disorders such as parasomnias or restless legs syndrome [38,39,40,41].

PSG is an effective way of diagnosing SB, but it is costly and requires specialized personnel and equipment. This makes it unfeasible for use in dental clinics and, in particular, in general dental practice. Therefore, in recent years, portable ambulatory instruments have been developed, providing information similar to PSG but more affordable and easier to handle. Its validity is still under discussion and requires further research, but it can be very useful as a clinical approach to SB evaluation [42]. EMG-EKG is a three-channel Holter-type device designed to detect the surface EMG signal of the two masseter muscles, and the HR by EKG. This EKG capability is what differentiates this device from other portable devices and supports its efficacy. The reliability of EMG-EKG has been proven with a very good diagnostic yield [3,43]. However, these studies have not been conducted in an OSA population. Additionally, there are many cases of undiagnosed OSA among patients.

The relationship between OSA and SB can vary based on the criteria used to measure muscle activity following apnea–hypopnea (AH) events [44,45]. This difference in criteria can lead to discrepancies in existing studies and may result in an overestimation of SB in patients with OSA. Given that OSA is often undiagnosed and frequently associated with SB, it is important to ensure that the ambulatory EMG used to measure muscle activity is reliable in such cases. It is necessary to exclude increased muscle tone following an apnea episode, which is part of the American Academy of Sleep Medicine (AASM) definition of arousal to prevent confusion [34]. 

This study aimed to compare the number of SB episodes in a population suspected of having OSA, as diagnosed by PSG and analyzed manually by a sleep expert, with that obtained manually and automatically by a portable EMG-EKG device (Bruxoff^®^). The objectives were to estimate the diagnostic validity of the EMG-EKG device for the diagnosis of SB, segment the sample based on the severity of OSA, and estimate the diagnostic validity of the EMG-EKG device for the diagnosis of SB in both manual and automatic analysis modes.

## 2. Results

During the recruitment period, forty-one patients underwent a full night of PSG with the simultaneous EMG-EKG device. In seven patients, the EMG-EKG device failed to record, and this information was excluded from the reliability analysis. Also, during the initial phase, we had problems with storage and methods of distinguishing between different recordings with the EMG-EKG device: eleven records (eleven participants) were removed from further analysis. One patient presented unusual cephalic movements, observed in the video, which could act as a confounding factor, so it was excluded from the final sample. Overall, records of 22 individuals (15 males and 7 females) with a mean age of 46.55 were accepted.

The descriptive sleep data (Table 1) show a sample of predominantly overweight patients (BMI= 25.0–29.9) with a minimum BMI of 17.93 and a maximum of 40.62.

The time of sleep stages is inside normal values except for the augmented proportion of the N1 stage (25.49 ± 16.32). The oximetry data mean values are compatible with a partial sleep apnea population pulse oximetry affecting values. The mean of sleep efficiency (81.18 ± 14.18) is the average value for the PSG testing in a sleep laboratory.

As shown in Table 2, the total events of SB per night in the PSG study were on average (8.41 ± 10.85) lower than those obtained with EMG-EKG device manual analysis (14.64 ± 10.76) and automatic (22.68 ± 16.02).

The tonic SB episodes were predominately against phasic SB episodes along the PSG and manual EMG-EKG analysis (Table 2).

The Spearman correlation between the apnea and hypopnea episodes and the SB episodes is negative (r = −0.402 (*p* = 0.06)) in the total of the sample, which means that when the number of apnea episodes increases, the number of SB episodes decreases with PSG recordings, but it is a non-significant correlation.

When we compared the variables of SB between the OSA (n = 16) and the non-OSA (n = 6) group, we obtained an increase in SB episodes from PSG analyses (13.71 ± 13.76) to manual (16.14 ± 10.73) and automatic (23.14 ± 11.69) EMG-EKG analyses in the non-OSA group, respectively. The mean number of SB episodes decreased from the non-OSA group to the OSA group with both PSG (5.93) and EMG-EKG analyses (automatic = 22.47, manual = 13.93). However, this decrease was minor in proportion compared to the automatic EMG-EKG analysis mode (from 23.14 to 22.47) (Table 3).

The phasic episodes were considerably lower in the OSA group with PSG analysis compared to the EMG-EKG results, and it is significant (Table 3). By segmenting the sample by the degree severity of OSA, the severe OSA patients were found to have fewer SB episodes than moderate or mild OSA patients with both PSG and EMG-EKG recordings, but it is not significant. The tonic episodes predominate against phasic episodes. The phasic episodes decreased considerably from non-OSA to OSA patients with PSG analysis compared to EMG-EKG analysis (Table 4).

**Figure 1:**
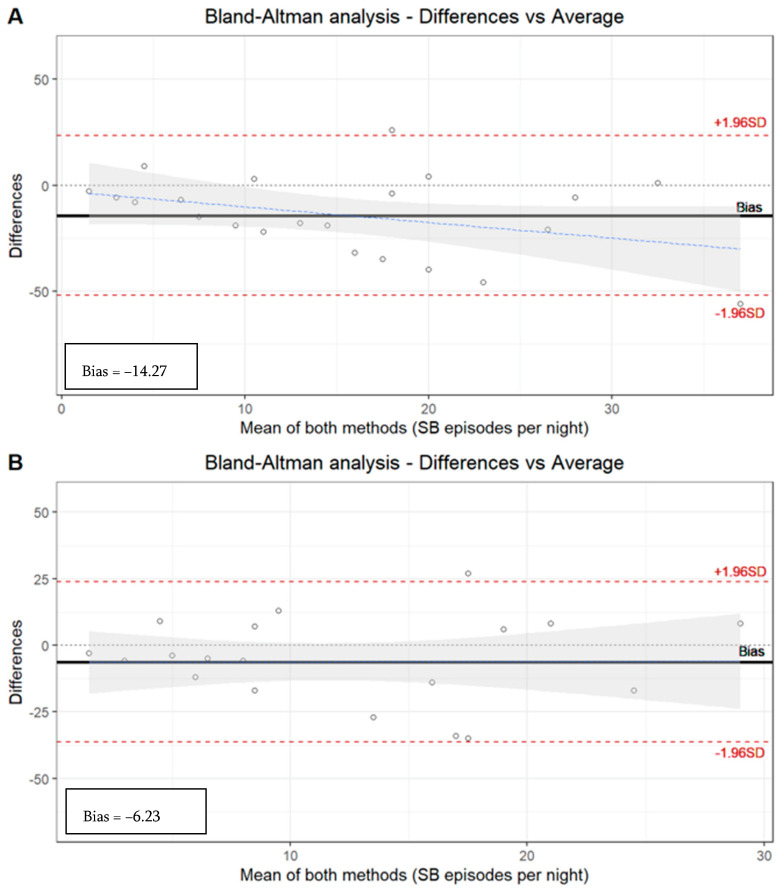
Bland–Altman analysis. (**A**) Manual analysis of PSG recordings versus automatic EMG-EKG device analysis. Limits agreement = +23.52, −52.07 (bias = −14.27). (**B**) Manual analysis of PSG recordings versus manual EMG-EKG device analysis. Limits agreement = +23.89, −36.34 (bias = −6.23).

Although the ICC [0.55 (*p* < 0.05)] based on the number of SB episodes in all the subjects along the three tools (PSG, manual EMG-EKG, and automatic EMG-EKG) shows a moderate correlation, a wide dispersion can be observed with the Bland–Altman representation (Table 5, Figure 1).

The EMG-EKG automatic analysis measures 14.27 units more than PSG, and the analysis denotes a proportional systematic bias, with a negative trend of the differences as the magnitude of the measured variable increases. The results with the manual EMG-EKG device analysis improved (measures 6.23 units more than PSG) but were not good (Figure 1).

The limits agreement of both EMG-EKG automatic and manual analysis is beyond the desirable limits of the S.D. The ICC based on the number of SB episodes in the segmented sample by severity degree of OSA along the three tools (PSG, manual EMG-EKG, and automatic EMG-EKG) shows an acceptable agreement in the non-OSA (0.61) and mild OSA (0.53, *p* < 0.05) groups. However, there is an insufficient ICC in the moderate (0.24) and severe (0.23) OSA groups (Table 5).

## 3. Discussion

It is important to note that this study is only the second one to compare the Bruxoff^®^ device to laboratory PSG. Additionally, it is the first study to compare both manual and automatic EMG-EKG analysis in an OSA population. The results showed that the diagnostic accuracy was acceptable for non-OSA and mild OSA patients. However, in patients with moderate or severe OSA, apneas could act as a confusing factor in the diagnosis of SB with an EMG-EKG portable device.

Some authors describe the possibility that there is a subtype of patients with subclinical or mild OSA that exhibit EMG activity corresponding to SB. This activity could play a protective role against OSA [28]. It is important to keep in mind that OSA and SB share structures that play a fundamental role in protective functions during sleep. Furthermore, there are inter-individual differences [46,47].

Therefore, it is essential to clarify the PSG criteria for the evaluation of SB and its comorbidities. This will help design quality studies and avoid biases in the evaluation [34,40,48]. Different authors suggest that SB studied with PSG in patients with OSA usually occurs close to AH events [22,27,48].

Comparative studies between EMG and PSG in the literature are limited and have low sample sizes. There have been only ten studies conducted so far, with sample sizes ranging from five to forty-nine participants [3,49,50,51,52,53,54,55,56,57]. Similar to our case, the limitation of sample size is a common issue. This can be attributed to the low prevalence of SB [16], the high cost of PSG, and the time required for both PSG and EMG analysis. These factors make it difficult to collect a large sample quickly, and there are often records that need to be discarded due to interference or failures.

In eight of the studies, different portable EMG devices were used [49,50,51,52,53,54,55,56], while two studies used the same EMG-EKG device that we used [3,57]. Yanez-Regonesi et al. compared the EMG-EKG device with the PSG laboratory but did not perform both automatic and manual analysis of the records [57]. Castroflorio et al. compared the portable device with PSG type II (without EEG) and excluded the AOS patients with questionaries [3]. In our study, we compared a portable EMG-EKG with PSG type I and performed manual PSG analysis. We also performed both automatic and manual EMG analysis, which could be marked as strengths of our design. Only two similar studies have separated the groups by OSA severity, like in our case, although with different designs [50,57].

Most studies have focused on a young population, typically between 21 and 28 years of age [3,52,53,56]. However, some authors, such as Mainieri [51] and Yanez-Regonesi [57], have used samples with mean ages similar to ours, which is between 41 and 50 years. In our sample, there are more men than women (15 men and 7 women out of a total of 22 participants). Only Yamaguchi’s study had an equal number of men and women (4 men and 4 women out of a total of 8 participants) [52], while Castroflorio et al.’s study had an almost equal number of men and women (12 women and 13 men out of a total of 25 participants) [3]. The condition we are studying, SB, does not differentiate between sexes, so the fact that our sample is not homogeneous between men and women should not create any bias. The prevalence of SB in adults is between 8% and 12% and decreases with age, dropping below 3% to 5% after the fifth decade [16,58]. Therefore, the medium age of the sample should not create any bias. However, OSA increases with age and is more prevalent in men, so it is a factor that needs to be considered when studying its association [23,59].

In our sample, the correlation between the apnea and hypopnea episodes and the SB episodes is negative in the total of the sample, which suggested that when the number of apnea episodes increases, the number of SB decreases in PSG recordings. Authors like Yap suggest that AH and SB events are probably epiphenomena in adult patients with coexisting OSA and SB, where SB events are predominantly featured after AH events and allude to a specific form of secondary SB triggered by sleep micro-arousals [60]. Nevertheless, we believe that this kind of activity could act as a confusion factor and should be considered as an AH final expected hypermotor activity rather than a secondary SB if there is not a minimum window of time between AH and the EMG hypermotor activity.

Yanez-Regonesi et al. found no association between AHI and the RMMA index, and they showed an acceptable diagnostic accuracy in terms of sensitivity (83.3%) and specificity (72.2%). However, they found a consistent and systematic difference in the measurement of SB episodes per hour of sleep between Bruxoff^®^ and PSG [57]. Castroflorio et al. found an excellent agreement with sensitivity and specificity of 91.6% and 84.6%, respectively. However, they used a PSG Type II as the gold standard and did not include OSA groups [3].

The accuracy of ambulatory devices used to detect sleep disorders depends on how well they correlate with the gold standard, which is PSG. It is crucial to improve the accuracy of automatic analysis of portable EMG devices to avoid overestimation of sleep disorders. In our study, the total events of SB per night recorded during PSG were lower than the number obtained from manual and automatic analysis of EMG-EKG devices. The mean number of SB episodes decreased from the non-OSA group to the OSA group in both PSG and EMG-EKG analyses. However, the decrease was smaller with automatic EMG-EKG analysis.

Our findings are consistent with the results of Martynowicz’s study, which found that the relationship between OSA and SB depends on the severity of OSA [45]. However, there are few studies on this relationship, and those that exist use different methods and have different goals. Okeson and Sjöholm did not find any differences in SB between OSA and non-OSA patients, but their sample was not segmented by the severity of OSA, and severe OSA patients were not included in the sample, respectively [30,31]. On the other hand, Okura suggests that OSA patients with SB have a unique phenotype of OSA and also emphasizes the distinct relationship of respiratory events with RMMA and non-specific masticatory activity (NSMA) [61].

In our sample, we found that the agreement between PSG and EMG-EKG devices is acceptable in non-OSA and mild OSA groups, but it is insufficient in moderate and severe OSA groups. We suspect that the exclusion or inclusion of the EMG event following the respiratory event (which we have discarded with PSG analysis) could explain the variability of the results. This could lead to an overestimation of SB in moderate and severe OSA patients when using EMG portable devices, especially when including that hypermotor activity. Another study, conducted by Saito, found a positive and significant correlation between OMA and AHI [24]. As Kato pointed out in 1999, the OMA activity may introduce a bias if it is not excluded from the neurophysiological analyses [62].

In our study, we found that tonic episodes were more common than phasic episodes. The number of phasic episodes decreased significantly in OSA patients compared to non-OSA patients with PSG analysis, as opposed to EMG-EKG analysis. Previous studies suggested that phasic episodes may have a protective role against OSA [28,59]. However, our study design only establishes a correlation between different instrumental tools and does not analyze the risk or protection factor. We obtained an acceptable ICC based on the number of SB episode accounts in all the subjects using the three tools (PSG, manual EMG-EKG, and automatic EMG-EKG). Other studies have obtained better diagnostic yield values, but these studies did not research the possible bias of OSA activity for SB estimations and found no association between the AHI and RMMA index [3,57].

Additionally, it is important to note that if the portable device is unable to identify the sleep stage, and an event fitting the criteria for RMMA occurs during wake time, it would be scored as an SB event. This could lead to an overestimation of SB [63], which in turn overestimates its association with other sleep disorders. Therefore, it is essential to complement the instrumental diagnosis of SB with clinical examination and the patient’s self-referred tests to assess the sequelae of SB [1].

The clinical consequence of SB is the true indicator of the need for treatment [42,64]. Therefore, definitive EMG ambulatory evaluation of SB should be increasingly implemented in the clinical setting, and not just in research, as it is the only reliable and objective measure to determine whether bruxing activity is present and active. Similarly, EMG is a useful tool for proper follow-up as a measure of the efficacy of certain therapeutic approaches. The use of EMG on a daily and reliable basis would mean being able to implement this tool in the same way that, for example, a periodontal chart is used for the staging of periodontal disease and its progression.

EMG-only devices may not have sufficient diagnostic yield for SB in populations in which OSA has not been previously ruled out. Therefore, the use of screening questionnaires such as STOP bang, and exploration of the oropharynx such as Mallampati class objectification, among other methods, could help guide whether it would be advisable to perform respiratory polygraphy in addition to EMG [19,41]. The combined use of respiratory polygraphy with EMG also allows for the complete screening of both entities (SB-OSA) and is also used for the follow-up of patients who use a mandibular advancement device. It would be interesting to use portable respiratory polygraphs that include EMG in masseters, like the one used by Winck [33]. Including masseter and temporalis muscle EMG montage in sleep units as routine would be useful to improve the knowledge about the relationship between SB and OSA.

Bruxism is a continuous activity, so it is important to have instruments that can record several nights in an unrestricted way, such as EMG, and refine them. Deregibus et al. demonstrated good reproducibility over time of the Bruxoff^®^ with no significant difference observed in the SB episodes per hour of sleep over three nights of recording [43]. Hence, determining new correlations and updated cut-off points is important [42].

All the EMG portable device designs and software should comply with the recommendations of the SENIAM project (Surface Electromyography for the Non-Invasive Assessment of Muscles), which has resulted in European recommendations for sensors and sensor placement procedures, and signal processing methods for surface electromyography (SEMG). The EMG-EKG portable device used for this study complies with those recommendations. However, not all EMG portable devices share a similar protocol [65,66,67]. Once the performance of portable EMG has been improved, it could be used for concordance studies against other types of novel tools that are emerging due to the evolution of technology, big data, and artificial intelligence [68,69,70]. Such studies would allow them to be performed longitudinally and more fluently than with PSG in a sleep lab.

In the case of studies on dental materials used in oral rehabilitation in bruxism patients, biases are significant, as SB is not objectively measured. By promoting the use of EMG, and encouraging clinicians and researchers in different fields of dentistry to utilize it more frequently, many biases can be prevented. For instance, biofeedback is already being used to manage SB with the help of EMG devices [53]. Further research in this area can lead to the development of non-invasive, reversible, and cost-effective methods for managing patients.

The findings indicate that manual analysis of SB events is more dependable than automatic analysis in our sample. Professionals who manage this type of patient would benefit from training and calibration in this type of analysis, as in the case of DC/TMD exploration for temporomandibular disorders [71].

It would be advisable to perform a basic OSA screening of all patients with suspected SB. In patients without OSA or with mild OSA, there is a reasonable concordance between the results of PSG manual analysis and those obtained by the EMG-EKG device with automatic and manual analysis for the SB diagnosis. However, in these patients, manual analysis of bruxing events with the EMG-EKG device shows greater reliability than automatic analysis.

### Limitations

A patient attending the sleep unit may suffer from “laboratory” effects on the first night, but it was not feasible for us to perform more than one night of PSG recording. However, previous studies have reported no overall first-night effect on the severity of RMMA frequency in patients with SB [72]. The groups are not balanced due to the low sample size, and there is a predominantly OSA population. We are collecting more data in this regard together with another hospital (multicenter study). The simultaneous placement of the surface electrodes of the portable device and PSG could generate interference and a poorer quality of signal reception. We tried to improve this limitation with smaller surface electrodes for the EMG-EKG device than those normally included in the package of the EMG and EKG electrodes.

## 4. Materials and Methods

Twenty-two (n = 22) participants underwent a full night of PSG testing (Deltamed Coherence 5.0 system) with simultaneous recording of the Bruxoff^®^ EMG-EKG device (OT Bioelettronica, Torino, Italy). Procedures were conducted following the STrengthening the Reporting of OBservational studies in Epidemiology (STROBE) statement and checklist [73]. The study protocol was approved by the Ethics Committee of the Hospital Clínico San Carlos in Madrid (C.P.–C.I. 14/380-E). Written informed consent was obtained from all participants, and all procedures were conducted according to the Declaration of Helsinki. Variables referring to the number of SB episodes and SB index (episodes/h), measured with both tools and analyzed in the manual and automatic modes, were compared. Masticatory muscle activity was scored according to published criteria [34,40]. After PSG testing, the sample was segmented by severity of OSA according to AASM criteria [34].

### 4.1. Sample Selection

The participants of the study are adult patients attending the Sleep Unit (Clinical Neurophysiology Department) of San Carlos University Hospital (Madrid, Spain) who underwent an earlier screening based on a suspicion of OSA and SB, the latter by self-referred bruxism tests (Paesani modified test) and physical examination [74]. For OSA, a neurophysiologist performed screening through anamnesis, anxiety–depression questionnaire, Epworth test, and additional examinations when other sleep disorders were suspected [41,75].

Exclusion criteria were major neurological disorders, psychiatric disorders, other sleep disorders, psychoactive medication, edentulism, or under 18 years of age. The clinical examination (tooth wear, masticatory muscle myalgia, temporomandibular joint arthralgia, hard tissue, soft tissue, and masseter and/or temporal hypertrophy) was performed according to diagnostic criteria for temporomandibular disorders (DC/TMD) and the American Academy of Orofacial Pain (AAOP) criteria and conducted by a dentist with ability in orofacial pain [71,76].

Finally, for the patients who did not meet the exclusion criteria and had a positive SB screening, a PSG diagnosis was performed by an experienced clinical neurophysiologist with specific training in SB. EMG-EKG with artifacts or other technical problems were excluded. The audio and video recordings were used to confirm the analysis [77]. As a result, a sample of 22 subjects with an average age of 46.55 ± 10.06 was achieved, comprising 15 men and 7 women. A concordance between the EMG-EKG portable device and the PSG (gold standard) design was used with six participants without OSA and sixteen with OSA. The sample of OSA patients was segmented by the degree of severity in three groups: Mild OSA = 7 (AHI = 5–14.9/h), Moderate OSA = 3 (AHI = 15–29.9/h), Severe OSA = 6 (AHI ≥ 30/h) [78].

### 4.2. PSG Recordings

The full-night monitoring recordings in the Sleep Laboratory (minimum of 8 h in bed) were performed using a Deltamed Coherence 5.0 system. PSG recordings were made according to the AASM recommendations [34], comprising six EEG derivations; right and left EOG; submental, masseter, and leg EMG; nasal cannula/pressure and oronasal thermal flow; thoracic and abdominal respiratory effort bands; snoring; body position sensor; pulse oximetry; audio and video recordings. Impedance values were checked and adjusted (<5 Ω), and standard calibrations were performed.

All PSG recordings were manually reviewed according to international criteria [34]. In the SB and OSA group, the diagnosis was confirmed by PSG performed by a sleep expert, following blind masking concerning the clinical examination. The AHI (episodes of AH per hour) was used to categorize OSA groups by severity level, according to published criteria [34,78].

#### PSG Sleep Bruxism Analysis

**Figure 2:**
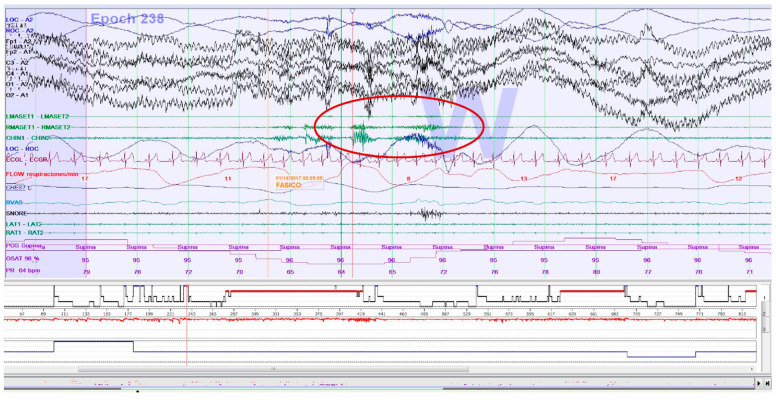
Epoch (30 s) of a polysomnography recording: an electromyographic phasic event of sleep bruxism.

SB events were estimated through rhythmic (RMMA; Figure 2) and non-rhythmic masticatory muscle activity (MMA) recorded with EMG on the masseter muscles (surface electrodes). Published criteria for SB episodes in PSG were followed [25]. The presence of > 4 RMMA-MMA/SB episodes/h was considered for the calculation of dichotomous variables. For the calculation of quantitative variables, the type of SB event is decided: phasic event (three or more EMG bursts, at least 0.25 s and up to 2.0 s; Figure 2), tonic event (at least one EMG burst > 2.0 s), and mixed event (both types) [40,79].

**Figure 3:**
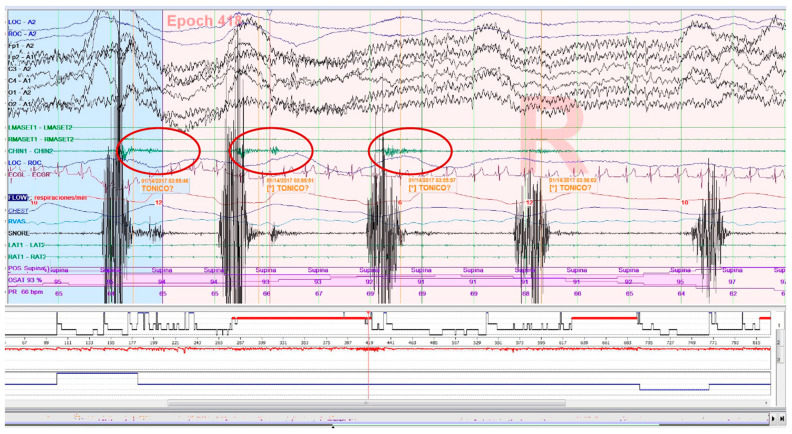
Epoch (30 s) of a polysomnography recording: tonic electromyographic episode, corresponding to sleep-related oromotor activity (OMA), following snoring. It is excluded as a sleep bruxism episode.

Increased muscle tone following an apnea episode, which is part of the AASM criteria definition of arousal [34], as well as sleep-related oromotor activity (OMA; Figure 3) different from RMMA-MMA/SB were excluded to avoid possible confounding bias. All isolated SB events, independent of respiratory events, were accepted according to EMG criteria, regardless of whether accompanied by arousals.

### 4.3. Bruxoff Sleep Bruxism Analysis

**Figure 4:**
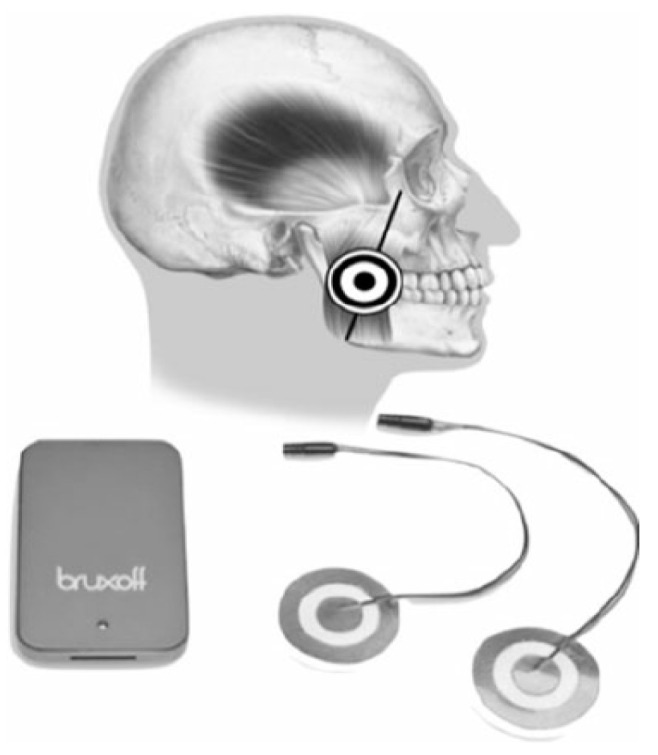
EMG-EKG electromyography and electrocardiography device.

Bruxmeter (version 2.0.2.4) is the software system of the EMG-EKG device (Bruxoff^®^; Figure 4 and Figure S1).

Interpretation is performed both manually, with the investigator analyzing the raw data, and automatically, with the device’s software analyzing the data to generate a diagnosis. According to data obtained in previous studies, automatic analysis reached a sensitivity of 91.6% [3]. The MicroSD card provided data for the diagnostic variables: bruxing event, number of bruxing events per hour of sleep (SB index), and number of bruxing events per night.

**Figure 5:**
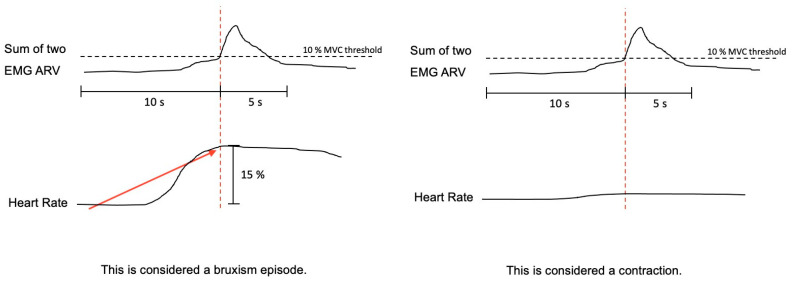
The algorithm used by Bruxmeter software to detect sleep bruxism episodes in manual mode, based on the autonomic activation cascade of sleep bruxism (SB), differentiating between SB episode and contraction, depending on the previous increase of the heart rate.

The bruxism event criteria depend on whether the analysis is performed in manual or automatic mode. Manual mode: EMG signal with peaks >0.25 s and an average amplitude of 10% of the patient’s maximum voluntary contraction (MVC), being preceded 1 s earlier by an increase in HR of 15%. Automatic mode: EMG signal with an amplitude of at least 10% of the patient’s MVC, preceded by an increase in HR of 20%, 1–5 s before (Figure 5).

### 4.4. Statistical Analysis

The variables used were the apnea–hypopnea index (AHI), SB Index, number of apnea events, number of hypopnea events, and number of SB events. Descriptive variables such as means and standard deviations were used. The sample passed the Shapiro–Wilk normality test. Continuous variables with a normal distribution were analyzed by the *t*-test. In addition, the sample was segmented according to the degree of severity of OSA and according to the types of SB episodes. The analysis of variance (ANOVA) between the non-OSA group and several degree severities of OSA was calculated. Spearman correlation for the apnea and hypopnea episodes and SB episodes was used. The Bland–Altman plot [80] was used to quantify the agreement between both methods (PSG and EMG-EKG). For Bland–Altman analysis, the program R Ver. 4.1.3 (R Foundation for Statistical Computing, Institute for Statistics and Mathematics, Welthandelsplatz 1, 1020 Vienna, Austria) was used. The concordance was calculated through the intraclass correlation coefficient (ICC). All calculations were performed with the SPSS v24.0 statistical package (SPSS Inc., Chicago, IL, USA). The *p*-values equal to or less than 0.05 were considered statistically significant.

## 5. Conclusions

In patients with moderate or severe OSA, apneas could act as a confusing factor in the diagnosis of SB with portable electromyography devices.

To differentiate the muscle activity that meets the criteria for SB from the muscle activity consecutive to the AH episode, it is recommended to clarify the analysis scores, particularly for the programming of portable device algorithms.

It would be beneficial to replicate studies with a similar design and expand the sample size to validate these findings. This would provide data to enhance the SB evaluation algorithms of portable devices for automatic analysis.

## Figures and Tables

**Table 1:** Descriptive sleep data of the sample.

	N = 22
	**Mean ± SD**
**Physical data**	
Age	46.55 ± 11.06
BMI	27.23 ± 5.38
**Sleep data**	
SPT (min)	411.55 ± 27.31
TST (min)	330.05 ± 62.42
SLT (min)	13.86 ± 26.87
Sleep efficiency (%)	81.66 ± 14.89
WASO (min)	56.30 ± 47.86
Awakes (number)	44.05 ± 25.16
**Sleep stage distribution**	
N1/SPT (%)	25.12 ± 16.60
N2/SPT (%)	43.85 ± 9.37
N3/SPT (%)	15.99 ± 10.91
R/SPT (%)	15.43 ± 5.92
**Pulse oximetry data**	
Mean (%)	93.45 ± 2.98
Max (%)	98.32 ± 1.04
Min (%)	81.09 ± 11.03
CT90 (%)	12.94 ± 23.65
**Sleep apnea data**	
No. apneas	106.18 ± 161.17
No. hypopneas	30.32 ± 31.24
No. apneas + hypopneas	136.50 ± 172.87
AHI	25.25 ± 32.83

BMI body mass index, SPT sleep period time, TST total sleep time, SLT sleep latency time, WASO wake time after sleep onset, CT90 total time lower 90% O2Sat, AHI apnea–hypopnea index.

**Table 2:** Data of sleep bruxism.

		N = 22
	**Mean ± SD**	**t**
**Polysomnography**		
No. episodes/night	8.41 ± 10.85	3.63
No. episodes/h	1.49 ± 2.05	3.39
No. phasic episodes	2.00 ± 4.48	2.09
No. tonic episodes	5.55 ± 7.06	3.68
No. mixed episodes	0.86 ± 1.67	2.42
**Automatic Bruxoff**		
No. episodes/night	22.68 ± 16.02	6.64
No. episodes/h	3.92 ± 2.71	6.78
No. phasic episodes	5.82 ± 5.37	5.06
No. tonic episodes	5.77 ± 6.90	3.87
No. mixed episodes	1.23 ± 1.87	3.06
**Manual Bruxoff**		
No. episodes/night	14.64 ± 10.76	6.37
No. episodes/h	2.54 ± 1.95	6.13
No. phasic episodes	5.27 ± 4.50	5.49
No. tonic episodes	8.05 ± 7.82	4.82
No. mixed episodes	1.32 ± 2.00	3.07

One-sample *t*-test was used for statistical analysis. Total SB events along the TST total sleep time, excluding the sleep-related oromotor Activity (OMA) with the EMG-EKG device and the gold standard (PSG, manual EMG-EKG, and sutomatic EMG-EKG). The significance level set as *p* < 0.05.

**Table 3:** Sleep bruxism data with the segmented sample.

			N = 22
	Non OSA ± SD N = 6	OSA ± SD N = 16	F
**SB Polysomnography**			
Total episodes	13.71 ± 13.76 *	5.93 ± 8.64 *	4.58
Phasic episodes	4.43 ± 7.39 *	0.87 ± 1.52 *	10.10
Tonic episodes	8.14 ± 8.57	4.33 ± 6.20	1.83
Mixed episodes	1.14 ± 1.86	0.73 ± 16.62	0.25
Ep./h	2.11 ± 2.07	1.20 ± 2.07	0.74
**SB Automatic Bruxoff**			
Total episodes	23.14 ± 11.69	22.47 ± 18.07	0.95
Phasic episodes	7.14 ± 6.25	5.20 ± 5.04	0.331
Tonic episodes	5.00 ± 4.65	6.13 ± 7.97	2.76
Mixed episodes	1.43 ± 1.81	1.13 ± 1.95	0.00
Ep./h	4.38 ± 2.38	3.70 ± 2.90	0.80
**SB Manual Bruxoff**			
Total episodes	16.14 ± 10.73	13.93 ± 11.08	0.20
Phasic episodes	6.43 ± 6.47 *	4.73 ± 3.39 *	5.07
Tonic episodes	8.57 ± 8.26	7 80 ± 7.89	0.03
Mixed episodes	1.14 ± 1.86	1.40 ± 2.13	0.18
Ep./h	3.15 ± 2.35	2.26 ± 1.74	1.19

An unpaired *t*-test was used for statistical analysis. Total SB events along the TST total sleep time, excluding the sleep-related oromotor Activity (OMA) with segmented sample (non OSA, OSA). The significance level was set as * *p* < 0.05.

**Table 4:** Sleep bruxism data with the segmented sample by OSA severity degree.

					N = 22
	Non OSA ± SD N = 6	Mild OSA ± SD N = 7	Moderate OSA ± SD N = 3	Severe OSA ± SD N = 6	F
**SB Polysomnography**					
Total episodes	16 ± 13.55	5.57 ± 6.13	10.33 ± 17.89	3.17 ± 4.66	1.83
Tonic episodes	9.50 ± 8.52	5.85 ± 2.21	6.67 ± 11.54	2.17 ± 2.86	1.18
Phasic episodes	5.17 ± 7.80	0.57 ± 1.13	1.67 ± 2.88	0.67 ± 1.21	1.52
Mixed episodes	1.33 ± 1.97	0.43 ± 0.78	2.00 ± 3.46	0,33 ± 0.82	0.97
Episodes/h	2.46 ± 2.03	0.93 ± 1.01	2.67 ± 4.61	0.80 ± 0.70	1.40
**SB Automatic Bruxoff**					
Total episodes	24.50 ± 12.19	26 ± 20.44	18 ± 24.26	19.33 ± 12.13	0.26
Tonic episodes	4.83 ± 5.07	6.71 ± 5.67	8 ± 13	4.50 ± 8.12	0.21
Phasic episodes	7.67 ± 6.68	7.29 ± 5.31	2.33 ± 3.21	4.00 ± 4.56	1.06
Mixed episodes	1.67 ± 1.86	0.86 ± 1.21	1.33 ± 1.52	1.27 ± 2.86	0.72
Episodes/h	4.68 ± 2.45	4.34 ± 3.24	2.6 ± 3.55	3.33 ± 2.19	0.50
**SB Manual Bruxoff**					
Total episodes	16.83 ± 11.58	16.14 ± 10.30	14 ± 18.19	11.00 ± 8.22	0.32
Tonic episodes	9 ± 8.96	8.29 ± 6.39	7.67 ± 11.59	7.00 ± 8.44	0.61
Phasic episodes	6.5 ± 7.09	6.71 ± 3.86	4 ± 2.64	3.00 ± 1.55	0.92
Mixed episodes	1.33 ± 1.96	1.14 ± 1.86	2.33 ± 4.04	1.00 ± 1.26	0.29
Episodes/h	3.33 ± 2.52	2.6 ± 1.41	2.03 ± 2.65	1.95 ± 1.71	0.55

One-factor ANOVA was used for statistical analysis. Total SB sleep bruxism events along the TST total sleep time, excluding the sleep-related oromotor activity (OMA) with segmented sample (non OSA, mild OSA, moderate OSA, and severe OSA) according to the AHI values. *p* > 0.05.

**Table 5:** Agreement data with the segmented sample by OSA severity degree.

			SB Episodes (N = 22)
Non OSA N = 6	Mild OSA N = 7	Moderate OSA N = 3	Severe OSA N = 6
0.61	0.53 *	0.24	0.23

Agreement, ICC intraclass correlation coefficient: total SB sleep bruxism episodes per night along the three tools (PSG, manual EMG-EKG and automatic EMG-EKG) with segmented sample by the degree severity of OSA obstructive sleep apnea. The significance level was set as * *p* < 0.05.

## Data Availability

The data presented in this study are available on request from the corresponding authors. The data are not publicly available due to ethical restrictions.

## Supplementary Materials

The following supporting information can be downloaded at https://www.mdpi.com/article/10.3390/clockssleep5040047/s1: Figure S1: EMG-EKG Device.

## Abbreviations

AASM:	American Academy of Sleep Medicine
AH:	Apnea-Hypopnea episode
AHI:	Apnea-Hypopnea Index
BMI:	Body Mass Index
CT90:	total time lower 90% O_2_Sat.
DC/TMD:	Diagnostic criteria for the Temporomandibular Disorders
EEG:	Electroencephalogram
EKG:	Electrocardiogram
EMG:	Electromyography
EOG:	Electrooculogram
HR:	Heart Rate
ICC:	Intraclass Correlation Coefficient
MMA:	Masticatory Muscle Activity
MVC:	Maximum Voluntary Contraction
OMA:	Sleep-related Oromotor Activity
OSA:	Obstructive Sleep Apnea
PSG:	Polysomnography
RMMA:	Rhythmic Masticatory Muscle Activity
SB:	Sleep Bruxism
SD:	Standar Desviation
SEMG:	Surface electromyography
SLT:	Sleep Time Latency
SPT:	Sleep Period Time
TST:	Total Sleep Time
WASO:	Wake time After Sleep Onset
