# Supplementary figures and images for: Diagnostic Accuracy of a Portable Electromyography and Electrocardiography Device to Measure Sleep Bruxism in a Sleep Apnea Population: A Comparative Study
Source: Clocks Sleep. 2023 Nov 20;5(4):717–33. doi: 10.3390/clockssleep5040047 (PMC10660473; doi:10.3390/clockssleep5040047)

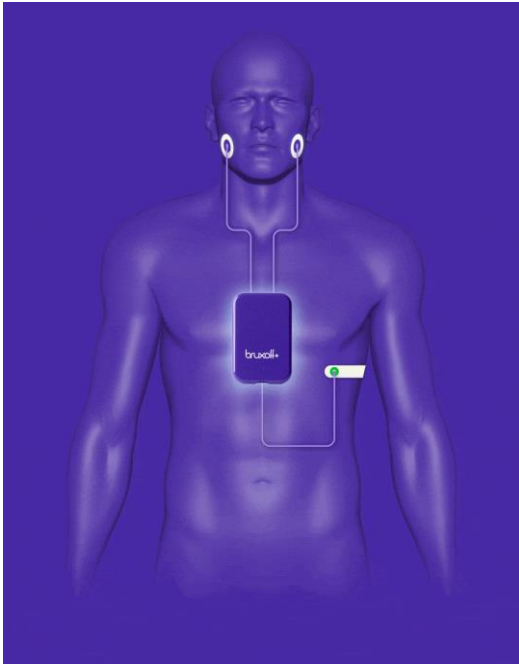

Figure S1. EMG-EKG Device (Bruxoff).

Supplement: Supplementary file 1 [file clockssleep-05-00047-s001.zip › clockssleep-2653680-supplementary.pdf]
